# Analysis of Statistically Predicted Rate Constants for Pyrolysis of High-Density Plastic Using R Software

**DOI:** 10.3390/ma15175910

**Published:** 2022-08-26

**Authors:** Rao Adeel Un Nabi, Muhammad Yasin Naz, Shazia Shukrullah, Madiha Ghamkhar, Najeeb Ur Rehman, Muhammad Irfan, Ali O. Alqarni, Stanisław Legutko, Izabela Kruszelnicka, Dobrochna Ginter-Kramarczyk, Marek Ochowiak, Sylwia Włodarczak, Andżelika Krupińska, Magdalena Matuszak

**Affiliations:** 1 Department of Physics, University of Agriculture Faisalabad, Faisalabad 38040, Pakistan; 2 Department of Mathematics and Statistics, University of Agriculture Faisalabad, Faisalabad 38040, Pakistan; 3 Department of Physics, COMSATS University Islamabad, Islamabad 45550, Pakistan; 4 Electrical Engineering Department, College of Engineering, Najran University Saudi Arabia, Najran 61441, Saudi Arabia; 5 Department of Pharmaceutical Chemistry, College of Pharmacy, Najran University, Najran 61441, Saudi Arabia; 6 Faculty of Mechanical Engineering, Poznan University of Technology, 60-965 Poznan, Poland; 7 Faculty of Environmental Engineering and Energy, Department of Water Supply and Bioeconomy, Poznan University of Technology, 60-965 Poznan, Poland; 8 Department of Chemical Engineering and Equipment, Poznan University of Technology, 60-965 Poznan, Poland

**Keywords:** pyrolysis of waste, high-density polyethylene, rate constant, numerical analysis, R software

## Abstract

The surge in plastic waste production has forced researchers to work on practically feasible recovery processes. Pyrolysis is a promising and intriguing option for the recycling of plastic waste. Developing a model that simulates the pyrolysis of high-density polyethylene (HDPE) as the most common polymer is important in determining the impact of operational parameters on system behavior. The type and amount of primary products of pyrolysis, such as oil, gas, and waxes, can be predicted statistically using a multiple linear regression model (MLRM) in R software. To the best of our knowledge, the statistical estimation of kinetic rate constants for pyrolysis of high-density plastic through MLRM analysis using R software has never been reported in the literature. In this study, the temperature-dependent rate constants were fixed experimentally at 420 °C. The rate constants with differences of 0.02, 0.03, and 0.04 from empirically set values were analyzed for pyrolysis of HDPE using MLRM in R software. The added variable plots, scatter plots, and 3D plots demonstrated a good correlation between the dependent and predictor variables. The possible changes in the final products were also analyzed by applying a second-order differential equation solver (SODES) in MATLAB version R2020a. The outcomes of experimentally fixed-rate constants revealed an oil yield of 73% to 74%. The oil yield increased to 78% with a difference of 0.03 from the experimentally fixed rate constants, but light wax, heavy wax, and carbon black decreased. The increased oil and gas yield with reduced byproducts verifies the high significance of the conducted statistical analysis. The statistically predicted kinetic rate constants can be used to enhance the oil yield at an industrial scale.

## 1. Introduction

Because of the increasing consumer demand for everyday items, the reliance on synthetic plastics is growing year after year. Plastics are well-known for their durability, chemical stability, and adaptability. These traits make them ideal materials for automotive, household appliances, medical equipment, horticulture, micro-foamed polymeric structures, etc. [1,2,3]. Plastics are a widely used material, resulting in a production rate of 355 million tons per year in mainland Asia and the European Union, which account for 19% and 50% of the market share, respectively [2]. Managing plastic garbage is becoming more difficult and has a negative impact on the environment [4]. Out of this bulk amount of waste, only 9% is recycled, 60% is dumped in landfills, and the remaining is burned [5,6]. Burning plastic waste and fossil fuels is wreaking havoc on human health and marine life [7]. Herein, it is imperative to find novel and environmentally friendly methods to manage or recycle plastic waste into value-added products and protect our environment and planet [8].

Based on the type of treatment process utilized and the byproducts extracted from it, all recycling processes are categorized. Plastics are heated in a chamber during the pyrolysis process, which turns them into flammable gases and liquids [9,10]. The plastic waste is changed into small constituents and then subjected to an endothermic heating process [11,12]. Depending on the heating method, pyrolysis can be either traditional or microwave-assisted [6]. It can be used to valorize biomass, wood, rubber, scuffle tires, coffee hulls, oil shells, and chemicals [12]. It is important to note that pyrolysis is a chemistry-intensive process. Systematic experimental and numerical tests are required to anticipate the physical and chemical properties of pyrolysis products. The lack of research on the statistical optimization of rate constants for effective pyrolysis of wastes, particularly plastic waste, is another factor supporting the aforementioned concerns.

By using appropriate statistical models, the process parameters must be statistically optimized for maximum output and good product selectivity. Therefore, it is critical to develop a model that simulates the pyrolysis of plastic, the most prevalent polymer, to determine the impact of operational parameters on pyrolysis efficiency. Using MLRM in R software, it is possible to statistically estimate the kind and quantity of basic products, including oil, gas, and waxes. As far as we are aware, no research publications on the statistical prediction of rate constants for the pyrolysis of plastics using MLRM analysis in R software have ever been published. Wirawan and Farizal [13] used a factorial design to optimize the pyrolysis process for plastic waste to produce fuel-grade products. They evaluated a 2 k factorial design for optimizing plastic-type, temperature, and residence time to maximize liquid product yield. The results demonstrated that the optimized pyrolysis operation could create diesel-like fuel at relatively low temperatures. The optimized temperature and residence time were reported as 175 °C and 180 min, respectively. The use of low temperature and long residence time was cited as the novelty of the work, as very few individuals had explored slow pyrolysis at such low temperatures [14]. Joppert et al. [14] used response surface methodology and factorial design to optimize the experimental conditions for the pyrolysis of mixed wastes. The optimization was done on process temperature reaction time and initial pressure. The tested models were discovered to be beneficial in providing knowledge to optimize experimental parameters that maximize the generation of predetermined liquid and gas components [15]. 

In another study, Krishna et al. [16] investigated the global dynamics of poly (methyl methacrylate), polystyrene, and ultra-high molecular weight polyethylene rapid pyrolysis. They used analytical Pyroprobe^®^ to generate data on isothermal mass loss for different time intervals in the range of 2–150 s. Integral reaction models were used to calculate the apparent pre-exponential factors and activation energies. This study predicted that fast pyrolysis of all three types of plastics would have low activation energies, demonstrating that the process of rapid pyrolysis is diffusion-limited. The time taken for the highest evolution of vapors was 12–45 s at a process temperature of 500 °C. The vapors evaluation time was reduced to 20–22 s at a process temperature of 600 °C. Harmon et al. [17] investigated the pyrolysis of plastic waste using a mechanistic model. A full and accurate description of the decomposition process demanded the solution of a huge system of equilibrium models due to the large number of species involved. The model predicted that as temperature increases, the olefin fraction breaks rapidly by increasing the gas proportion. When temperature and residence time increase, the production of aromatics also shows an increasing trend. However, it was stressed that the primary utility of the model depends on the accurate prediction of operational factors but not on the computation of the number of separate species under specific operating circumstances. Senneca and Tucciullo [18] used lumped kinetics scheme for the pyrolysis of n-hexadecane and n-decene compounds. They used the CDF model to investigate the chemical kinetics, which is crucial in hybrid propulsion because the mixture of gas products generated by pyrolysis impacts the combustion process. A complete pyrolysis procedure for high-density plastic was designed and verified from the published data. The study focused on the likelihood of producing soot rather than the specific yield of all products and reaction intermediates. A reaction network with 198 species and 6307 reactions was condensed into a very basic five-reaction mechanism on the basis that the primary tar produced by the pyrolysis of plastic waste is largely made up of aliphatics that can undergo progressive aromatization to polycyclic aromatic hydrocarbons and soot. The results of this study can not be generalized since the primary focus was on the production of tar from plastics.

We are pioneers in testing R software to evaluate a multiple linear regression model (MLRM) to forecast the amount of oil, gas, and wax produced by the pyrolysis of plastics. To the best of our knowledge, no one has previously published a study on how to use MLRM analysis in R software to statistically forecast the kinetic rate constants for plastics pyrolysis. The temperature-dependent rate constants were experimentally fixed at 420 °C in this investigation. For the pyrolysis of high-density plastic, rate constants with differences of 0.02, 0.03, and 0.04 from empirically set values were investigated. The anticipated improvements in the final products were also analyzed using a second-order differential equation solver in MATLAB. The key research aim was to identify a combination of statistically predicted kinetic rate constants that may have a significant role in increasing the efficiency of oil production at a commercial scale. This method enables the monitoring of the formation of individual products during pyrolysis by offering insight into the process of the production of specific products. The regression model results were statistically significant, having a positive correlation between the dependent and predictor variables. A lot of research work needs to be done to explore the sensitivity of the kinetic rate constants in assessing the efficiency of the individual kinetic rate reactions to get better results.

## 2. Statistical Prediction of Rate Constants

The experimental rate constants were obtained from the literature [19]. Eidesen et al. [19] used these rate constants to estimate the amount of oil and gas from the thermal pyrolysis of HDPE. Our study used open-source R software to predict the rate constants statistically. The predicted rate constants were used in MATLAB simulation of the thermal pyrolysis of HDPE. The effect of these rate constants on the products, like oil, gas, and waxes was elaborated, and the findings of the simulation work were compared with the experimental data to suggest the best combination of rate constants for high selectivity and yield.

The experimental rate constants were taken as dependent variables, and the values derived statistically were used as independent variables. After applying the model, the correlation between the response and predictors was confirmed using 2D and 3D graphs. The general form of MLR Equation (1) was then used individually in R software for each rate constant. Z represents our predicted rate constant or statistical rate constant, α and β are MLR equation coefficients, X and Y are the differences between experimental and predicted rate constants, and n = 1, 2, and 3 are the number of statistical operations.

Z_n_ = A_o_ + α_n_X_n_ + β_n_Y_n_ (1)

**Figure 1:**
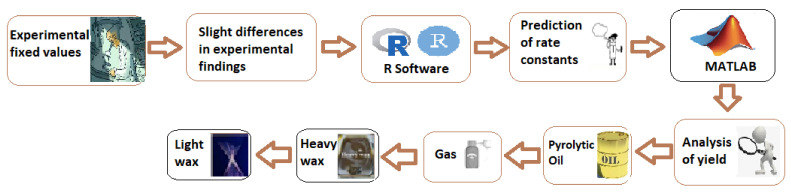
Graphical illustration of study on the rate constants for pyrolysis of high-density plastic.

The statistically determined rate constants are then employed in MATLAB by using the second-order differential equation solver to evaluate the percentage efficiency in yield at 420 °C. Figure 1 provides the flow of the conducted research work. 

The general equation for the multiple linear regression model (MLRM) is Z = A_0_ + αX + βY. This model is used in R software to examine the relationship between dependent and independent variables. MLR equation is used in R software to estimate the coefficient of regression for an outcome and a risk factor X, as well as an outcome and a hypothetical confounder Y. The computed statistical coefficients of regression are used in MLRM to generate a logical combination of rate constants. Three MLRM analyses with a difference of 0.02, 0.03, and 0.04 from the fixed experimental rate constant were performed at 420 °C [19]. MLR equation for each analysis is:

Z_1_ = A_0_ + α_1_X_1_ + β_1_Y_1_, Z_2_ = B_0_ + α_2_X_2_ + β_2_Y_2,_ and Z_3_ = C_0_ + α_3_X_3_ + β_3_Y_3_. (2)

Here, Z_1_, Z_2,_ and Z_3_ are dependent variables, and X_1,_ X_2_, X_3_, Y_1_, Y_2_, and Y_3_ are independent or predictor variables. The α_1_, α_2_, α_3_, β_1_, β_2_, and β_3_ are the coefficients of the MLR equation. The values of all the parameters are used in R software to predict an adequate combination of rate constants K_1_, K_1_, and K_3_. In analysis with a difference of 0.02, α_1_ = −0.04 and β_1_ = 4.56 × 10^−^^1^ are the coefficients of the MLR equation, and A_0_ = 5.35 × 10^−^^1^ is the intercept which is calculated by the MLR equation in R software. The coefficients X_1_ and Y_1_ are statistically significant as the value of *p* is less than 0.001, as marked in Table 1 with the symbol ‘***’. 

**Figure 2:**
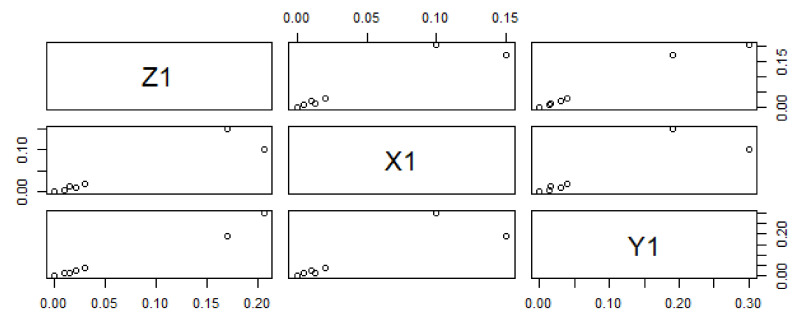
Data trending plot between dependent variable Z_1_ and predictor variables X_1_ and Y_1_ with a difference of 0.02 in experimentally fixed value at 420 °C.

**Figure 3:**
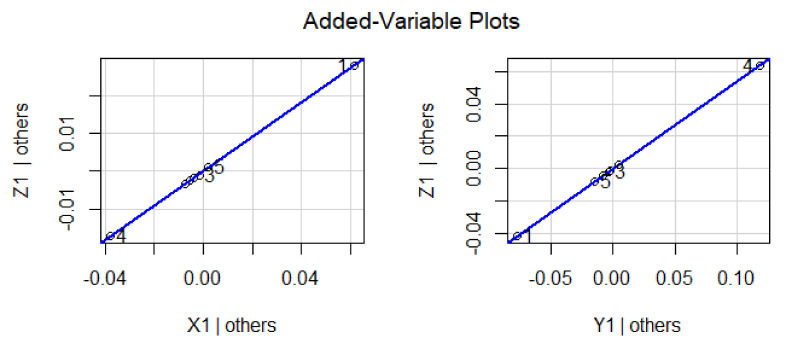
Representation of correlation among the dependent and predictor variables for a difference of 0.02 in experimentally fixed value at 420 °C.

**Figure 4:**
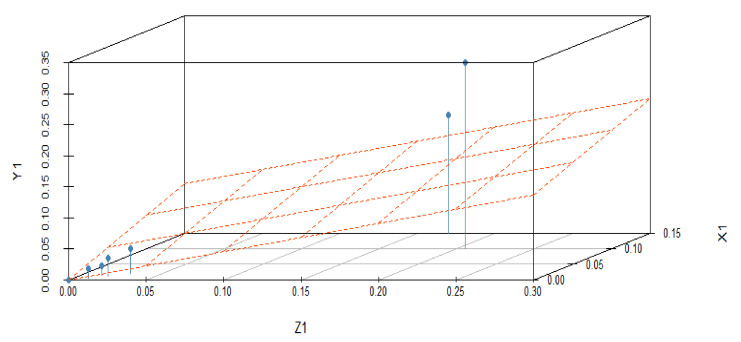
3D illustration of correlation among the dependent and predictor variables for a difference of 0.02 in experimentally fixed value at 420 °C.

The effect of variations in predictor variables on the dependent variables suggested that the regression coefficient α_1_ shows 0.456 units increment for Z_1_ when Y_1_ is held to be constant. Similarly, the regression coefficient β_1_ shows 0.534 units increment for Z_1_, when X_1_ is held constant. The dependent variable Z_1_ with predictor variables X_1_ and Y_1_ explores the nature of the data, as shown in Figure 2. In this graphical illustration, the trend of dependent and predictor variables varies from bottom to top, which identifies that the results are significant due to a *p*-value of 0.001 and shows a positive relationship among the variables. The y-axis depicts the response variable, while the x-axis represents a single predictor variable. In Figure 3, the blue line describes a relationship between the predictor variable and the response variable when all other predictor variables are kept constant. The same trend can be verified by investigating the vertical blue lines in a 3D plot in Figure 4. After operating MLRM in R software, a suitable combination of rate constants (K_1_) is obtained, as listed below in Table 2. 

The coefficients of MLRM for the experimentally fixed rate constant at 420 °C with a difference of 0.03 and 0.04 are reported in Table 3 and Table 4, respectively. MLR equation for this analysis is:

Z_2_ = B_0_ + α_2_X_2_ + β_2_Y_2_ and Z_3_ = C_0_ + α_3_X_3_ + β_3_Y_3_.

Here, α_2_ = 4.56 × 10^−1^, α_3_ = 1.00, β_2_ = 5.35 × 10^−1^, and β_2_ = −4.51 × 10^−9^ are the coefficients of MLRM while B_0_ = −3.55 × 10^−5^ and C_0_ = 4.00 × 10^−2^ are the intercepts calculated using MLRM in R software. The X_2_ and X_3_ are significant because the *p* values are revealed to be <0.001, but Y_2_ and Y_3_ are not statistically significant as the value of *p* > 0.001 as shown in Table 3 and Table 4 with the symbol “***”. 

**Figure 5:**
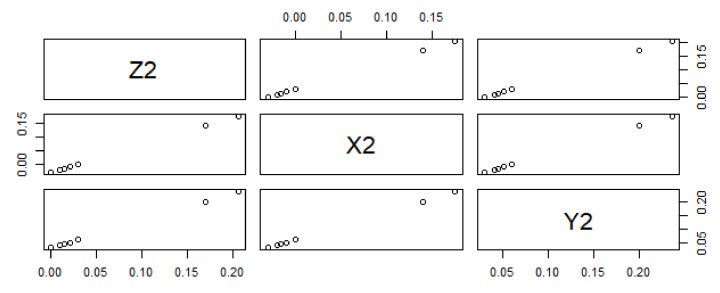
Data trending plot between dependent variable Z_2_ and predictor variables X_2_ and Y_2_ with a difference of 0.03 from the experimentally fixed values at 420 °C.

**Figure 6:**
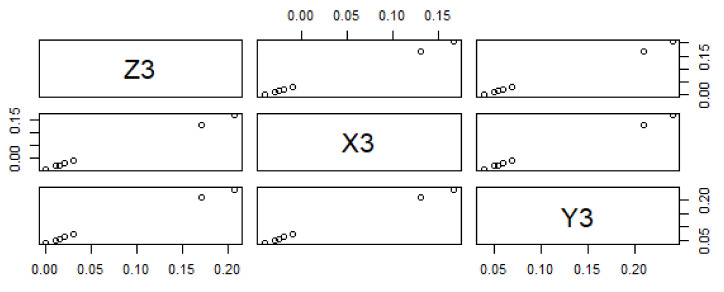
Data trending plot between dependent variable Z_3_ and predictor variables X_3_ and Y_3_ with a difference of 0.04 from the experimentally fixed value at 420 °C.

The reported data reveal a negative correlation between predictor variables Y_2_ and Y_3_ and responses Z_2_ and Z_3_. The data trend of dependent and predictor variables is significant for X_2_ and X_3_, while Y_2_ and Y_3_ are not statistically significant because the data trend does not exactly change from bottom to top, as shown in Figure 5 and Figure 6. The statistically predicted rate constants with a difference of 0.03 and 0.04 are mentioned in Table 5 and Table 6. 

**Figure 7:**
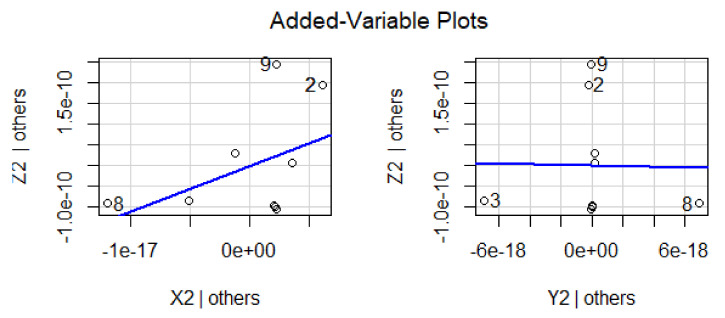
Representation of correlation among the dependent and predictor variables for a difference of 0.03 from the experimentally fixed value at 420 °C.

**Figure 8:**
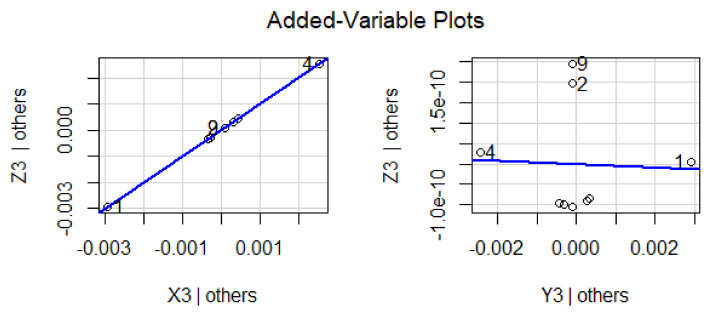
Representation of correlation among the dependent and predictor variables for a difference of 0.04 from the experimentally fixed value at 420 °C.

**Figure 9:**
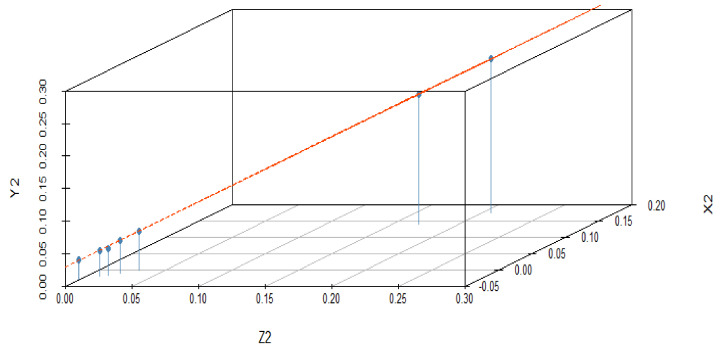
3D illustration of correlation among the dependent and predictor variables for a difference of 0.03 from the experimentally fixed value at 420 °C.

**Figure 10:**
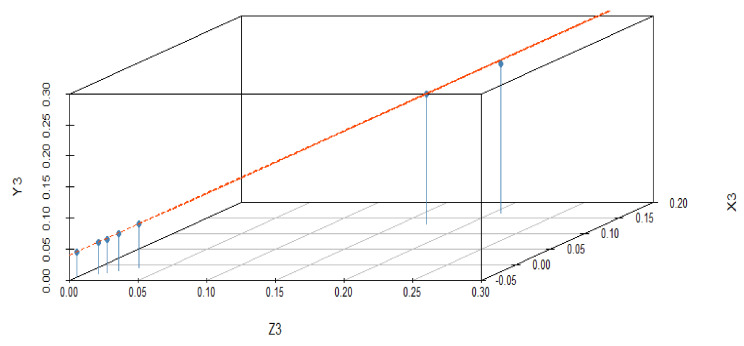
3D illustration of correlation among the dependent and predictor variables for a difference of 0.04 from the experimentally fixed value at 420 °C.

The response variable is given along the y-axis, while a single predictor variable is given on the x-axis. In variable-added plots, the blue line confirms the positive correlation for X_2_ and X_3_, while a negative correlation is evident for Y_2_ and Y_3_ because the *p*-value is revealed to be <0.001 and >0.001, respectively, as shown in Figure 7 and Figure 8. A similar pattern of such correlations is also confirmed from the vertical blue lines in 3D plots, as shown in Figure 9 and Figure 10.

The experimentally fixed (Z) and predicted rate constants (K_1_, K_2_, and K_3_), obtained by statistical analysis, are listed in Table 7. Both experimentally fixed and statistically predicted rate constants were solved through an ODE, such as [t, x] = ode23s (@reaction, time, C0) in MATLAB to investigate the effect of rate constants on percentage yield over process time. Here, t is time, X is percentage yield, and C0 represents the initial condition or number of moles. 

## 3. Effect of Statistically Predicted Rate Constants on Yield Concerning Process Time

The model Equations (3)–(7) were solved in MATLAB using the 23 s solver for ordinary differential equations (ODEs) (R2020a) [19]. In Equations (3)–(7), $\frac{dS}{dt}$ Indicates the mass rate at which HDPE is utilized, S is the mass of HDPE, HW is the mass of heavy wax, and Table 7 contains the values of experimental and statistical reaction constants (k_1_ to k_9_) [20,21,22].

(3) $$\frac{ds}{dt}=-k_{1}S-k_{2}S-k_{3}S-K_{4}S$$

(4) $$\frac{dHW}{dt}=K_{4}S-K_{8}\mathrm{Hw}-K_{9}\mathrm{HW}$$

(5) $$\frac{dLW}{dt}=K_{1}S-K_{6}\mathrm{Lw}-K_{5}\mathrm{LW}$$

(6) $$\frac{doil}{dt}=K_{2}S+K_{5}\mathrm{Lw}+K_{9}\mathrm{HW}-k_{7}\mathrm{Oil}$$

(7) $$\frac{dGas}{dt}=K_{3}S+K_{6}\mathrm{Lw}+K_{7}\mathrm{Oil}+k_{8}\mathrm{HW}$$

When the conditions for pyrolysis are met, the dominant products are LW with k_1_, oil with k_2_, gas with k_3_, and HW with k_4_ rate constants. Furthermore, the free radicals degrade LW to oil (k_5_ rate constant), LW to gas (k_6_ rate constant), and HW to gas (k_8_ rate constant) and oil (k_9_ rate constant). It was also evident that some parts of the oil changed to gas with reaction constant k_7_. Table 7 shows the values of the reaction constants ‘k’ at 420 °C.

**Figure 11:**
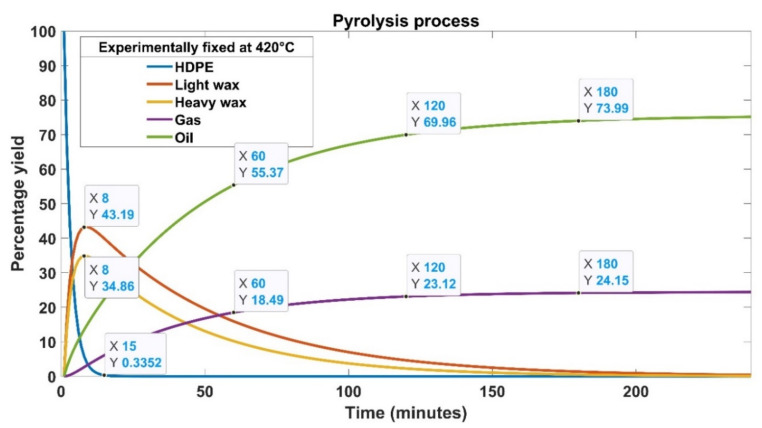
Graphical illustration of product type and yield for experimentally fixed rate constants at 420 °C.

**Figure 12:**
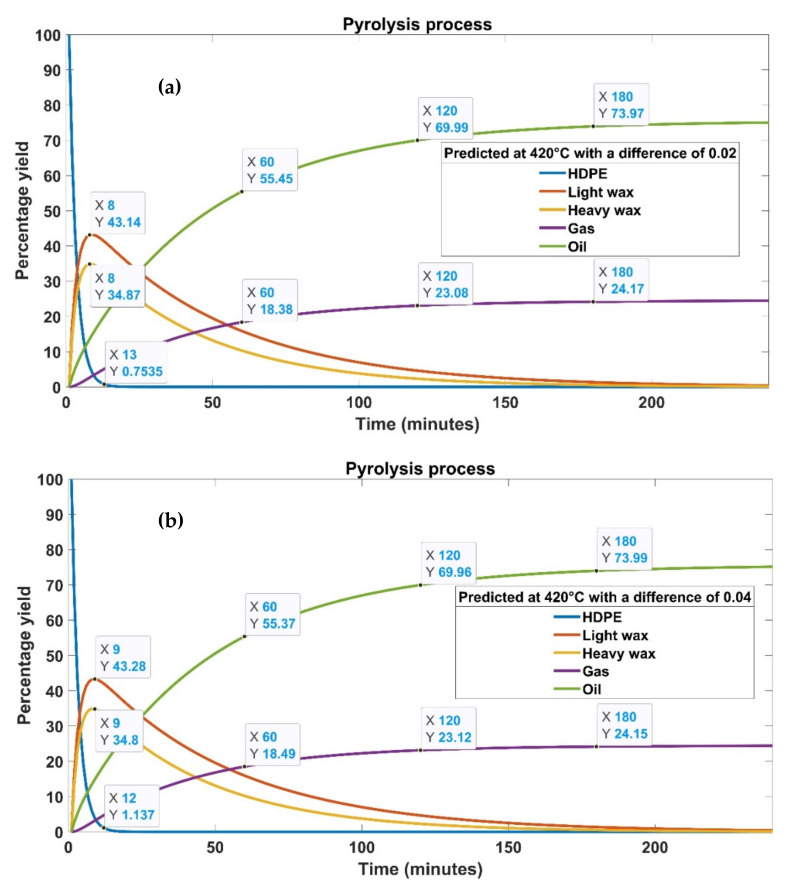

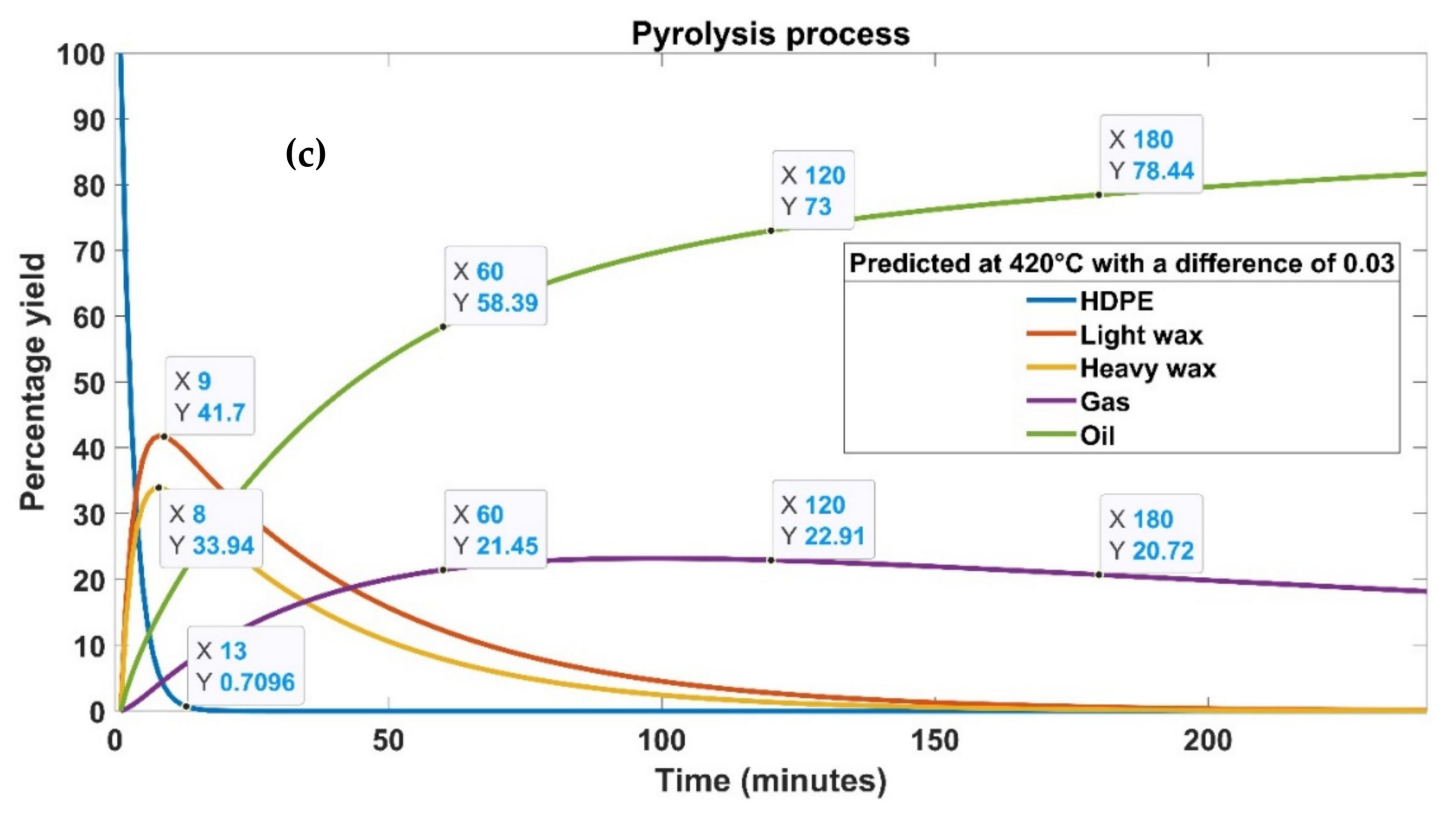
Graphical illustration of product type and yield for statistically predicted rate constants with a difference of (**a**) 0.02, (**b**) 0.03, and (**c**) 0.04 from the experimentally fixed value.

This analysis was carried out at a fixed temperature of 420 °C to explore the impact of experimental and statistical rate constants on yield over process time. HDPE is transformed into a variety of different organic compounds, including light and heavy waxes before the process reaches a stable state. A number of other substances are found in the reaction product mixture but in relatively low concentrations [23]. Aromatics, kerosene, and paraffin account for the majority of the waxes. Some of these waxes may continue to disintegrate further into smaller molecules as a result of the high temperature, resulting in carbon black. This carbon black is mostly made of carbon and contains very small particles. Carbon black is incredibly stable and will not react any longer. The experimentally determined and statistically predicted results demonstrate that HDPE degrades rapidly over time for the suggested temperature. A small portion of HDPE (0.33%) remained after 15 min of the pyrolysis process, as shown in Figure 11 and Figure 12. Figure 11 shows the product type and yield graphically for experimentally fixed rate constants at 420 °C. The trend of the products, obtained with the predicted rate constants with a difference of 0.02, 0.03, and 0.04 from the experimentally fixed value, is reported in Figure 12. Abbas and Shubar performed pyrolysis of high-density plastic [24]. It was discovered that higher cracking temperatures and longer reaction periods resulted in greater gas and coke output. The greater the temperature, the more aromatics are produced, resulting in lighter oil with reduced viscosity. 

According to our findings, both experimental and statistical procedures entail first breaking down HDPE into smaller particles, which then change into heavy and lighter waxes. When light and heavy waxes produced during the conversion process are consumed, oil and gas output decreases significantly [20]. The cracking of HPDE and production of oil, liquid, gas, and waxes are nearly the same in all cases except for the rate constant difference of 0.03. The product yield differs significantly for a 0.03 difference in rate constants. Expect this case, both experimental and statistically predicted rate constants produced nearly 73% to 74% oil, 24% gas, and 2% amorphous solid [24]. The oil yield must be in the 70–80% range for these findings to be acceptable. In this investigation, once the pyrolysis process was completed, 78% of the oil was recovered with a rate constant difference of 0.03. This amount is approximately 5% greater than the oil produced in other cases, showing a high recovery rate of the tested statistical model. However, gas yield remained slightly lower (21%) in this case. The solid residue was about 1% compared to other cases, which produced 2% solid residue. The light and heavy waxes were almost zero in all cases at the end of the process.

Table 8 details the time-dependent transformation of HDPE into oils, gases, and waxes. After 60 min of pyrolysis time, the rate constants with a difference of 0.03 produced the highest gas and oil yield of 22% and 58%, respectively. After 120 min of the pyrolysis process, all trials produced the same amount of gas (23%); however, oil yield remained 3% higher for a difference of 0.03 than all other cases. The gas yield dropped to 21% from 23% after 180 min of the processing time carried out with a difference of 0.03. On the other hand, all other cases showed a slight increase in gas yield from 23% to 24%. The most significant difference was observed in oil yield, which jumped from 73% to 78% after 180 min with a difference of 0.03. Numerous works have been done to investigate the behavior of the effects of operational parameters on the production of oil yield [25,26,27,28,29,30,31,32]. Uzun et al. [27] used a fixed bed reactor to study the co-pyrolysis of synthetic and PS waste at 500 °C through a semi-batch process. The highest oil yield was reported to be about 65% on the completion of the process. The best reaction kinetics with the highest liquid yield was achieved during pyrolysis of PS/HDPE at a ratio of 1:2. When compared with oil produced from a single bench biomass pyrolysis, the bio-oil obtained via co-pyrolysis offers better characteristics. Carbon and hydrogen concentrations increased, whereas oxygen concentrations declined with the processing time. The bio-oils of co-pyrolysis showed high calorific values, making them an environmentally friendly fuel. The inclusion of HDPE in co-pyrolysis enhances the amount and quality of oil in terms of the dispersion of hydrocarbons. 

A comparison of the percentage yield of oil using different wastes, temperatures, and methods is provided in Table 9. Salem et al. [28] thermally decomposed HDPE in a batch-type reactor. They reported an oil yield of 70% at a temperature of 550 °C. The experimental findings were also modeled using synthetic reaction kinetics of HDPE degradation. In a two-stage process, the kinetic parameters of the primary stage of decomposition of HDPE showed high activation energy, while the second stage of decomposition produced gases, liquids, and solid fractions due to the intramolecular hydrogen shift and termination step. The reported model may be used to construct commercial plastic waste management facilities that use thermal processes to generate fuel and energy. Khan et al. [29] conducted pyrolysis of HDPE waste to obtain oil, gas, and char. The high heating and longer reaction times minimized char production. The lower temperatures created volatile chemicals. They reported 77.03% oil after 2 h of pyrolysis time in the temperature range of 330 °C to 490 °C. These findings suggest that the physicochemical properties of liquid fuels may be exploited by changing process time and temperature. Rodríguez-Luna et al. [30] pyrolyzed HDPE in the temperature range of 450–550 °C. The characterization of wax and oil products showed the formation of alkenes, alkanes, and dienes. The intermolecular hydrogen transformation and scissions were the possible reasons for the formation of these moieties. The oil contained a wider range of compounds with shorter chain lengths than wax. A design of experiments was used to determine the best conditions for maximizing the volatile fraction. After 30 min of treatment time, a volatile fraction corresponding to 97% of HDPE mass was produced at 500 °C.

Park et al. [31] reported 80% oil at a temperature of 730 °C in two-step pyrolysis of PVC waste. Although they claimed a little higher oil yield (2%) than our study, the temperature employed to generate these results was substantially higher. The high temperature limits the commercial impact of the work because recycling plastics at such temperatures is not a cost-effective approach. Sun et al. [32] used sludge char as a catalyst to produce oil and aromatic oil from the pyrolysis of plastic waste. Product type and yield were influenced by the catalytic temperature, residence duration, and feedstock compositions. When the catalytic temperature was 600 °C and the residence period was 1 s, the selectivity of the catalyst toward monocyclic aromatics was reported to be up to 75.3%. Styrene and xylene content in oil reached 29.1% and 12.5%, respectively. When the temperature was raised to 800 °C by keeping the residence time of 1 s, the selectivity of catalyst to bicyclic aromatics was up to 64.4%. At this temperature, naphthalenes accounted for 47.5% of the oil product. In their investigation, the interaction between polypropylene, polyethylene, and polystyrene raised the bicyclic aromatic selectivity from 46.8% to 53.7%.

Gracida-Alvarez et al. [33] studied the effect of process temperature on the pyrolysis of HDPE. In a two-step micropyrolysis reactor, the pyrolysis of HDPE generated vapors that were then subjected to secondary degradation by increasing the temperature and vapor residence time. The dispersion of the product was significantly impacted by both vapor residence time and temperature. At 625 °C, a wide range of liquid and gas products were produced with a vapor residence time of 1.4 s. For a process temperature of 675 °C and vapor residence time of 5.6 s, mostly mono-and-poly aromatics and hydrocarbon gases were produced. Miandad et al. [34] pyrolyzed plastic waste into oil and char. The plastic trash was transformed into liquid oil at 420 °C for 75 min. High aromatic components in oil make it unsuitable as fuel unless it is improved by distillation, refining, or blending with diesel. The same temperature was chosen for the presented work to keep the process cost-effective. Relatively better oil yield at moderate temperature was achieved by employing an appropriate statistical model in R software and finding the best combination of rate constants.

## 4. Conclusions

It is concluded that developing statistical models to simulate plastic waste recycling through pyrolysis is the need of the present time. Statistical models are important in determining the impact of operational parameters on the process efficiency and selectivity of the products. In this study, the rate constants for pyrolysis of HDPE were statistically predicted using MLRM analysis in R software. Such analysis has never been reported in the published literature. A low temperature of 420 °C was chosen to predict the rate constants for a cost-effective pyrolysis process. MLRM in R software was applied to these rate constants that differed by 0.02, 0.03, and 0.04 from empirically determined values. Depending on the predictor variables, the dependent variable altered the product yield. The oil output increased from 78% to 88% with a 0.03 variation from the experimental fixed rate constants, but light wax, heavy wax, and carbon black decreased. The production of oil, liquid, gas, and waxes was nearly the same in all cases except for the rate constant difference of 0.03. The product yield differed significantly for a 0.03 difference in rate constants. On completion of the pyrolysis process, 78% of the oil was recovered with this rate constant difference. This amount was approximately 5% greater than the amount of oil produced in other cases, showing a high recovery rate of the tested statistical model. The gas yield dropped to 21% from 23% at the end of the process. The rest of the trials revealed a slight increase in gas yield from 23% to 24%. The most significant difference was observed in oil yield, which jumped from 73% to 78%. These findings suggest the high significance of the tested statistical model at low pyrolysis temperature. Future work should be focused on the optimization of the pyrolysis process for plastics by choosing multiple temperature-dependent activation energies and pre-exponential factors.

## Figures and Tables

**Table 1:** Coefficients of MLRM for the experimentally fixed rate constant at 420 °C with a difference of 0.02. Where ‘***’ shows a statistically significant value.

	Estimate	Stand. Error	*t*-Value	*p*-Value
Intercept	−3.549 × 10^−5^	7.15 × 10^−5^	−0.496	0.637
X_1_	4.564 × 10^−1^	2.439 × 10^−3^	187.139	1.57 × 10^−12^ ***
Y_1_	5.346 × 10^−1^	1.240	431.226	1.57 × 10^−14^ ***

**Table 2:** The rate constant (K_1_) was predicted using a dependent variable (Z_1_), and independent variables X_1,_ and Y_1_ with a difference of 0.02 in experimentally fixed value at 420 °C.

Dependent Variable Z_1_	Independent Variable X_1_	Independent Variable Y_1_	Rate Constant K_1_
0.17	0.15	0.19	1.70 × 10^−1^
2.43 × 10^−8^	2 × 10^−8^	2.9 × 10^−8^	−3.55 × 10^−5^
0.0301	0.02	0.04	3.05 × 10^−2^
0.206	0.1	0.3	2.06 × 10^−1^
0.0146	0.013	0.016	1.45 × 10^−2^
0.0104	0.005	0.015	1.03 × 10^−2^
2.25 × 10^−14^	2.00 × 10^−14^	2.50 × 10^−14^	−3.55 × 10^−5^
0.0205	0.01	0.03	2.06 × 10^−2^
3.48 × 10^−10^	2.00 × 10^−10^	5.00 × 10^−10^	−3.55 × 10^−5^

**Table 3:** Coefficients of MLRM for the experimentally fixed rate constant at 420 °C with a difference of 0.03. Where ‘***’ shows a statistically significant value.

	Estimate	Stand. Error	*t*-Value	*p*-Value
Intercept	3.000 × 10^−2^	2.074 × 10^−9^	1.446	2 × 10^−16^ ***
X_2_	1.000	3.507 × 10^−8^	2.852 × 10^7^	2 × 10^−16^ ***
Y_2_	2.724 × 10^−8^	3.494 × 10^−8^	7.800 × 10^−1^	0.465

**Table 4:** Coefficients of MLRM for the experimentally fixed rate constant at 420 °C with a difference of 0.04. Where ‘***’ shows a statistically significant value.

	Estimate	Stand. Error	*t*-Value	*p*-Value
Intercept	4.000 × 10^−2^	3.168 × 10^−9^	1.263 × 10^7^	2 × 10^−16^ ***
X_3_	1.000	3.919 × 10^−8^	2.552 × 10^7^	2 × 10^−16^ ***
Y_3_	−4.505 × 10^−9^	3.990 × 10^−8^	−1.130 × 10^−1^	0.914

**Table 5:** Multiple linear regression model with a difference of 0.03 from the experimental fixed values at 420 °C. Where ‘***’ shows a statistically significant value.

Dependent Variable Z_2_	Independent Variable X_2_	Independent Variable Y_2_	Rate Constant K_2_
0.17	0.14	0.2	1.71 × 10^−1^
2.43 × 10^−8^	2.4 × 10^−8^	2.46 × 10^−8^	2.31 × 10^−3^
0.0301	1.00 × 10^−4^	0.0601	3.21 × 10^−2^
0.206	0.176	0.236	2.06 × 10^−1^
0.0146	1.54 × 10^−2^	4.46 × 10^−2^	1.68 × 10^−2^
0.0104	1.96 × 10^−2^	4.0 × 10^−2^	1.26 × 10^−2^
2.25 × 10^−14^	2.22 × 10^−14^	2.28 × 10^−14^	2.31 × 10^−3^
0.0205	9.5 × 10^−3^	5.0 × 10^−2^	2.26 × 10^−2^
3.48 × 10^−10^	3.45 × 10^−10^	3.51 × 10^−10^	2.31 × 10^−3^

**Table 6:** Multiple linear regression model with a difference of 0.04 from the experimental fixed value at 420 °C. Where ‘***’ show a statistically significant value.

Dependent Variable Z_3_	Independent Variable X_3_	Independent Variable Y_3_	Rate Constant K_3_
0.17	0.14	0.2	1.70 × 10^−1^
2.43 × 10^−8^	2.39 × 10^−8^	2.47 × 10^−8^	2.38 × 10^−8^
0.0301	1.00 × 10^−4^	0.0601	3.01 × 10^−2^
0.206	0.176	0.236	2.06 × 10^−1^
0.0146	4.0 × 10^−4^	0.0446	1.46 × 10^−2^
0.0104	9.6 × 10^−3^	4.0 × 10^−4^	1.04 × 10^−2^
2.25 × 10^−14^	2.21 × 10^−14^	2.29 × 10^−14^	−1.80 × 10^−10^
0.0205	9.5 × 10^−3^	5.0 × 10^−4^	2.05 × 10^−2^
3.48 × 10^−10^	3.44 × 10^−10^	3.52 × 10^−10^	−1.80 × 10^−10^

**Table 7:** Both experimentally fixed and statistically estimated rate constants for pyrolysis of HDPE.

Experimentally Fixed Z	Predicted at 0.02 K_1_	Predicted at 0.03 K_2_	Predicted at 0.04 K_3_
k__1_ = 0.17	k__1_ = 1.70 × 10^−1^	k__1_ = 1.71 × 10^−1^	k__1_ = 1.70 × 10^−1^
k__2_ = 2.43 × 10^−8^	k__2_ = −3.55 × 10^−5^	k__2_ = 2.31 × 10^−3^	k__2_ = 2.38 × 10^−8^
k__3_ = 0.0301	k__3_ = 3.05 × 10^−2^	k__3_ = 3.21 × 10^−2^	k__3_ = 3.01 × 10^−2^
k__4_ = 0.206	k__4_ = 2.06 × 10^−1^	k__4_ = 2.06 × 10^−1^	k__4_ = 2.06 × 10^−1^
k__5_ = 0.0146	k__5_ = 1.45 × 10^−2^	k__5_ = 1.68 × 10^−2^	k__5_ = 1.46 × 10^−2^
k__6_ = 0.0104	k__6_ = 1.03 × 10^−2^	k__6_ = 1.26 × 10^−2^	k__6_ = 1.04 × 10^−2^
k__7_ = 2.25 × 10^−14^	k__7_ = −3.55 × 10^−5^	k__7_ = 2.31 × 10^−3^	k__7_ = −1.80 × 10^−10^
k__8_ = 0.0205	k__8_ = 2.06 × 10^−2^	k__8_ = 2.26 × 10^−2^	k__8_ = 2.05 × 10^−2^
k__9_ = 3.48 × 10^−10^	k__9_ = −3.55 × 10^−5^	k__9_ = 2.31 × 10^−3^	k__9_ = −1.80 × 10^−10^

**Table 8:** Process time-dependent product yield produced with experimentally fixed and statistically predicted rate constants.

Experimentally Fixed			Statistically Predicted		
Time (min)	Species	% Yield	% Yield at 0.02	% Yield at 0.03	% Yield at 0.04
60	Light wax	0	0	0	0
	Heavy wax	0	0	0	0
	Gas	19	18	22	19
	Oil	55	55	58	55
120	Light wax	0	0	0	0
	Heavy wax	0	0	0	0
	Gas	23	23	23	23
	Oil	70	70	73	70
180	Light wax	0	0	0	0
	Heavy wax	0	0	0	0
	Gas	24	24	21	24
	Oil	74	73	78	73

**Table 9:** A comparison of percentage yield of oil using different waste, temperatures, and methods.

Waste Type	Method	Temperature (°C)	Yield (%)	References
PS/HDPE	Co-pyrolysis	500	65	[25]
HDPE	Pyrolysis	550	70	[26]
HDPE	Pyrolysis	330–490	76	[27]
HDPE	Pyrolysis	450–550	77	[28]
HDPE	Two-step Pyrolysis	730	80	[29]
Mix	Pyrolysis	800	53	[30]
HDPE	Pyrolysis	535–675	57	[31]
PP, PE	Pyrolysis	420	80	[32]
HDPE	Pyrolysis	420	78	Current study

## Data Availability

The published data is available from the authors on a reasonable request.
